# EFFECT OF ACTIVITY-BASED INTERVENTION ON QUALITY OF LIFE AMONG OLDER ADULTS LIVING IN COMMUNITY

**DOI:** 10.1093/geroni/igae098.3341

**Published:** 2024-12-31

**Authors:** Jisu Seo, Rhayun Song, Yuelin Li, Eunna Oh

**Affiliations:** Chungnam National University, Daejeon, Republic of Korea; Chungnam National University, Daejeon, Republic of Korea; Chungnam National University, Daejeon, Republic of Korea; Chungnam National University, Daejeon, Republic of Korea

## Abstract

With age, older people experience reduced participation in activities. According to the Activity theory of aging, older adults can age successfully by engaged in their favorite activities. This study was conducted to systematically review RCTs that applied activity-based interventions (ABIs) to older adults living in community, and was to classify the ABIs into interaction-based social activities, exercise-based physical activities and self-directed individual activities, as well as compare and analyze the size of the effect on quality of life based on three types of activities. The analysis included 76 randomized studies from 6 Korean databases as well as 5 English databases. Cochrane’s Risk of Bias tool was used to assess the quality of the risk of bias. A CMA version 4 was used to calculate the effect size and a 95% confidence interval using a random effect model and to assess heterogeneity and publication bias. A network meta-analysis was conducted using R version 4.3.1 software’s “netmeta” package. Using GRADE, we assessed the quality of the evidence. ABIs applied to older adults living in community significantly improved their quality of life (SMD=0.56), and all three types of activity-based interventions significantly improved their quality of life. According to the result of ranking of network meta-analysis, self-directed individual activities was found to have the highest probability of being selected at 91.7%. As older people spend more time alone as aging, it is important to enhance their quality of life by encourage them to choose and participate in activities according to their own interests.

